# Chronic stress alters neurotransmitter co-expression and disrupts context discrimination in a sex-dependent manner

**DOI:** 10.1016/j.ibneur.2026.01.003

**Published:** 2026-01-06

**Authors:** Christopher Mazon, Ryan Betters, Gabriella Salmeron-Ceballos, Anthony Tomaziefski, Cristina Coffman, Renae Simonson, Elif Tunc-Ozcan

**Affiliations:** a Department of Neurosciences, University of New Mexico, Albuquerque, NM 87131, USA; b Biomedical Sciences Graduate Program (BGSP), University of New Mexico, Albuquerque, NM 87131, USA

**Keywords:** Chronic stress, Depression, Co-transmission, Context discrimination, Medial septum

## Abstract

Stress contributes to neuropsychiatric disorders by altering brain circuits and neurotransmitter signaling, often in a sex-dependent manner. The specifics of these stress-induced changes and their role in the development and perseverance of conditions like depression are largely unknown. We examined how context discrimination and neurotransmitter co-expression in the medial septum (MS) changes in response to unpredictable chronic mild stress (uCMS). Female mice subjected to uCMS showed significant context discrimination impairments compared to female controls, while male mice showed no differences in context discrimination as a result of uCMS. We conducted immunolabeling in the MS and found that in females, uCMS reduced the number of cholinergic (ChAT+) neurons while increasing the percent of neurons co-expressing ChAT & GAD67 (marker for GABAergic neurons). These changes suggest a link between chronic stress, neurotransmitter phenotype plasticity in the MS, and hippocampal dysfunction. These differences were observed in the absence of changes to apoptosis and overall neuron number and were specific to female mice; no significant changes to MS neurotransmitter expression was observed in males. Our future work will focus on further dissecting the specific molecular mechanisms behind these changes.

## Graphical Abstract

**Figure:**
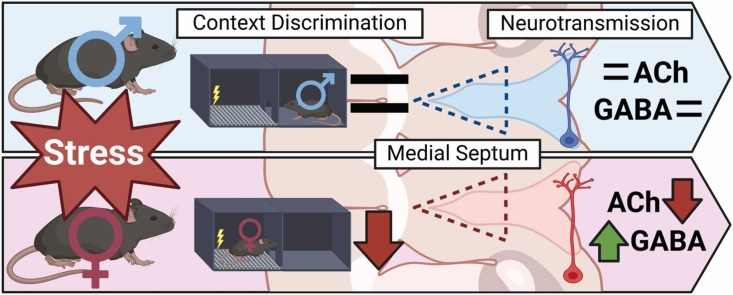

## 1 Introduction

Cognitive impairments are a defining feature of many neuropsychiatric disorders, including anxiety, depression, post-traumatic stress disorder (PTSD), and bipolar disorder (Dossi et al., 2020, Trivedi, 2006, Lima et al., 2018, Patel et al., 2016). Among these, hippocampal-dependent cognitive deficits are overrepresented and are strongly influenced by stress, a key environmental risk factor for these conditions (Agid et al., 2000). Despite the central role of the hippocampus in cognition and its vulnerability to stress, the upstream modulatory circuits that contribute to stress-induced hippocampal dysfunction remain poorly understood. One such candidate is the medial septum, which extensively innervates the hippocampus and play a critical role in modulating hippocampal-dependent learning and memory (Dannenberg et al., 2015, Kniffin et al., 2024, Mamad et al., 2015; Müller and Remy, 2018; Teles-Grilo Ruivo and Mellor, 2013; Wander et al. 2023; Paul et al. 2015).

Medial septum cholinergic and GABAergic projections are central to synchronizing hippocampal oscillations (Dannenberg et al., 2015, Kiraly et al., 2023, Vandecasteele et al., 2014, Yoder and Pang, 2005, Gu and Yakel, 2022, Smythe et al., 1992) and modulating neuronal plasticity (Mamad et al., 2015, Teles-Grilo Ruivo and Mellor, 2013, Smythe et al., 1992, Roland et al., 2014). GABAergic inputs from the medial septum are particularly critical for maintaining the excitation-inhibition balance within hippocampal circuits (Wander et al. 2023; Tóth et al., 1997; Sans-Dublanc et al. 2020). Disruptions in these pathways have been directly linked to impairments in hippocampal-dependent behaviors (Mamad et al., 2015, Wander et al., 2023, Sans-Dublanc et al., 2020, Niewiadomska et al., 2009, Dalrymple-Alford, 1994), further emphasizing the critical role of the medial septum in regulation of hippocampal function. Stress rapidly activates the medial septum (Gilad, 1987), and chronic stress has been shown to impair hippocampal learning and memory through disruptions in cholinergic neuromodulation (Kniffin et al., 2024, Tomar et al., 2021). Notably, depletion of medial septum cholinergic neurons exacerbates stress-induced spatial memory deficits (Craig et al. 2008), while neither stress nor cholinergic depletion alone produces these effects, highlighting the essential role of medial septum cholinergic neurons in mitigating the cognitive effects of chronic stress. Together, these findings underscore the medial septum as a critical node linking stress reactivity to hippocampal circuit integrity. Its widespread projections to distinct hippocampal subregions enable region-specific modulation of circuit excitability, rhythmicity, and plasticity. However, the specific neurotransmitter mechanisms through which medial septum neurons adapt or fail under chronic stress conditions, and how these changes influence hippocampal function and behavior, remain unknown. Addressing this gap is essential for identifying circuit-level processes that link stress to cognitive impairment and for uncovering potential therapeutic targets in neuropsychiatric disorders.

Recent studies show that cholinergic neurons can synthesize and co-express both acetylcholine (ACh) and γ-aminobutyric acid (GABA) (Saunders et al., 2015a, Granger et al., 2016). This property of the cholinergic neurons makes dual neurotransmitter signaling possible across multiple brain regions, including the frontal cortex (Saunders et al., 2015b, Granger et al., 2020), hippocampus (Takacs et al. 2018), entorhinal cortex (Desikan et al. 2018), striatum (Lozovaya et al. 2018), substantia nigra (Le Gratiet et al. 2022), and retinal ganglion cells (Sethuramanujam et al. 2016). Given this wide range and reach of effects, the cholinergic system has long been a focus of drug development for treating brain disorders; however, the co-expression of ACh-GABA in the cholinergic neurons has never been considered in these studies (Yu et al., 2014; Cummings et al. 2019; Mineur et al., 2009; Mineur et al. 2011; Rollema et al. 2009). Recent findings show that neurotransmitter phenotype plasticity profoundly impacts behaviors relevant to neuropsychiatric disorders. For instance, acute stress induces a lasting switch in dorsal raphe serotonergic neurons from glutamate to GABA co-transmission, causing generalized fear in mice (Li et al. 2024). In the hypothalamus, seasonal light changes trigger a switch between dopamine and somatostatin expression, and blocking this switch induces anxiety and depression-like behavior in rats (Dulcis et al. 2013). Interestingly, dopamine neurotransmitter switching in response to photoperiod changes is also observed in humans and is believed to contribute to seasonal affective disorder (Aumann et al. 2016). These findings illustrate the dynamic nature of neurotransmitter phenotype in response to environmental and physiological stimuli. Despite its therapeutic relevance, ACh-GABA co-expression remains an unexamined dimension of cholinergic signaling in stress-related dysfunction. Clarifying whether chronic stress drives maladaptive ACh-GABA co-expression changes in medial septum neurons and whether these changes contribute to hippocampal-dependent cognitive deficits provides a clear mechanistic rationale for the present study.

We hypothesize that chronic stress alters ACh-GABA co-expression within medial septum neurons, leading to cognitive impairments consistent with those seen in neuropsychiatric disorders. To test this hypothesis, we investigated how unpredictable chronic mild stress (uCMS) alters neurotransmitter identity in the medial septum and the resultant effects on hippocampus-associated context discrimination. Our findings demonstrate that uCMS disrupts both ACh-GABA co-expression in medial septum neurons and hippocampus-dependent behavior in a sex-dependent manner, exclusively affecting females.

## 2 Methods and materials

### 2.1 Animals

All animal procedures were approved by the University of New Mexico HSC Institutional Animal Care and Use Committee, and were performed in accordance with the Public Health Service Policy on Humane Care and Use of Laboratory Animals. All animals were housed 3–5 per cage on a 12-hour dark/12-hour light cycle in a controlled environment and received food and water *ad libitum*. All behavioral testing was conducted during the light period.

Eight to 10-week-old, naive C57Bl/6 male and female mice (Jackson Laboratories, Bar Harbor, ME, USA, JAX #204708) were used for all experiments, where animals were randomly assigned to experimental groups (control or uCMS) for 21 days. At least three separate cohorts of mice were run for all experiments. Animal numbers are provided in figure legends. Sample sizes were determined based on our previous studies using the same experimental design and behavioral endpoints, which consistently yielded robust effect sizes and reproducible results. No formal statistical power analysis was performed. The study included the following group sizes: control female (n = 7), uCMS female (n = 9); control male (n = 8), uCMS male (n = 7), totaling 31 mice.

### 2.2 Unpredictable chronic mild stress (uCMS)

Mice were subjected to various unpredictable chronic mild stressors for two weeks (Nollet, 2021). Before the start of any stressful stimulus, animals were transferred from the cage rack to a biological safety cabinet in the housing room or to a designated behavior room used for uCMS manipulations and exposed to two of the stressors listed below each day. Stressors were performed on a randomized schedule, and each stress duration varied depending on the type: between 10 min and 4 h, except for light cycle disturbances. At the end of each daily stress period, animals were replaced into clean cages and returned to the housing rack. Control animals were handled only for cage changes and behavioral testing.

1. Tilted cage: Home cages were tilted by approximately 45° against a sturdy object for the duration of the test.
2. Social stress: Mice were transferred from their home cage to an empty temporary cage then to a cage previously occupied by neighboring mice.
3. Light cycle disturbance: Animals were exposed to regular room light during the night cycle, or the lights were turned off during the day cycle.
4. Predator smells: A filter paper soaked with 1 μl of 10 % 2,4,5-Trimethylthiazoline (a component of fox urine/feces and the most used synthesizable reagent for inducing innate fear in rodents) was placed into the home cage and mixed around with the bedding.
5. Damp Bedding: The home cage bedding was soaked in approximately 150 mL of clean water.
6. No Bedding: All bedding from the home cage was removed.
7. No bedding + water: Animals were placed in a temporary cage without bedding and approximately 150 mL of clean water.

### 2.3 A-B discrimination test

**Fig. 1:**
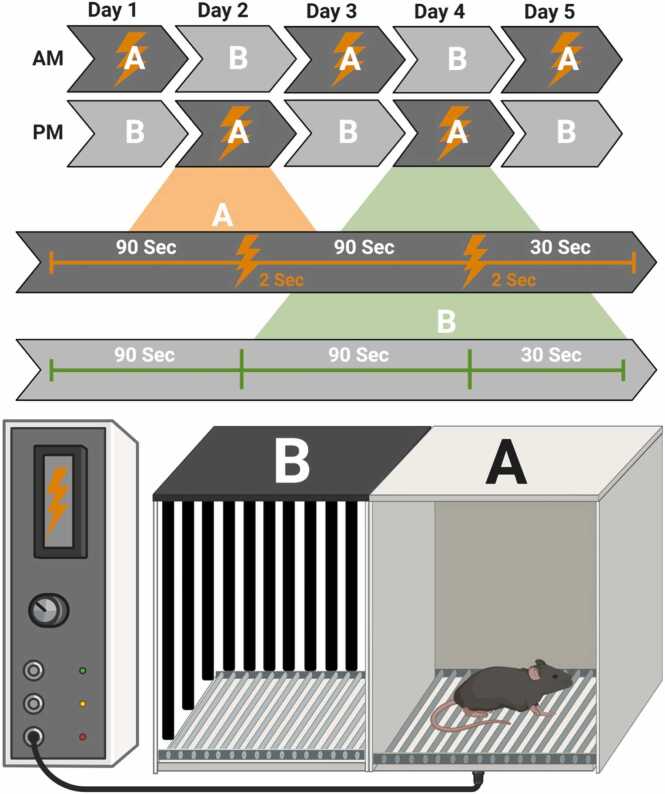
Graphical Schematic of the A-B Discrimination Test. Mice are subjected to either Context A (shock) or Context B (no shock-neutral); mice in Context A are exposed to two, 90-second acclimation periods, each followed by a 2-second foot shock (0.8 mA). Mice in Context B underwent the same protocol duration but without receiving any aversive stimulus. These contexts were reversed for each mouse, each day for five days, to evaluate any deficiencies in context discrimination.

The A-B Context Discrimination Test was conducted over five consecutive days, with each day consisting of two sessions (Fig. 1). During the first session in the morning, each mouse was placed individually in one of two distinct chambers: Context A (shock) or Context B (no shock). Mice in Context A were exposed to a 90-second acclimation period followed by a 2-second foot shock (0.8 mA). After a subsequent 90-second interval, a second identical foot shock was delivered, and mice were removed from the chamber 30 s later. Mice in Context B underwent the same protocol duration but without receiving any aversive stimulus. During the second session in the afternoon, mice were exposed to a second identical training session, except that the mouse initially subjected to Context A (shock) was subjected to Context B (no shock), and vice versa. This sequence was repeated once per day for five consecutive days, with the order of testing in Context A vs. Context B reversed every day for each individual mouse. Context A was a standard chamber with a stainless-steel floor, clear Plexiglas front wall, and aluminum side and back walls. Context B was a similar chamber, except the floor was a plastic non-shock floor, with side and back walls covered in striped black and white contact paper. All trials were recorded using a digital camera mounted above each chamber and data were collected using VideoFreeze™ Video Fear Conditioning Program (Med Associates Inc.). The duration of freezing behavior exhibited by each mouse in the two contexts was analyzed to assess their ability to discriminate between them, as freezing time directly correlates with this ability. A discrimination index, calculated as (freezing time in Context A - Context B) / (freezing time in Context A + Context B) before shock, was used to quantify discrimination performance, with higher scores reflecting enhanced behavioral functioning and greater ability to distinguish between the two contexts.

A-B Discrimination Test was performed at the University of New Mexico-Health Science Center Preclinical Core of the Center for Brain Repair and Recovery. Mice were transferred to the testing room 2 h prior to testing for acclimation to the test environment. The behavioral apparatus was wiped with 70 % ethanol prior to each trial and between trials. Behavioral analyses were performed by a single experimenter, blind to the experimental condition. All mice were sacrificed two days following the final training session for histological analysis.

### 2.4 Tissue collection and processing

Mice were transcardially perfused with cold perfusion buffer (500 mL 1X PBS, 0.005 g (5 mg) Heparin, 0.5 g Procaine) followed by cold 4 % paraformaldehyde solution (Sigma-Aldrich, #1.00496.0700). Brains were fixed overnight in 4 % paraformaldehyde solution and then transferred to 30 % sucrose for 24 h. Forty-micron floating sections were obtained using a cryostat (Thermo Scientific, Cryostar NX50) and preserved in cryoprotectant (125 mL Glycerol, 125 mL Ethylene Glycol, 250 mL 1X PBS) at −20° Celsius until further analysis.

### 2.5 Immunohistochemistry

Tissue sections were removed from cryoprotection and placed into 1X PBS in a black Netwell™ Reagent Tray, washed in PBS-T (1X PBS, 0.1 % Tween-20) 3 × 5 min, permeabilized in 0.4 % Triton X-100 for 20 min, blocked in 10 % normal donkey serum in 1 % BSA-PBS solution for 1 h at room temperature, and incubated overnight at 4°C in primary antibody diluted in 0.5 % BSA-PBS. The next day, sections were washed in PBS-T 3 × 5 min, incubated in a fluorophore-conjugated secondary antibody (Alexa Fluor) diluted in 0.5 % BSA for 90 min at room temperature, and rinsed in PBS-T 3 × 5 min. Following one final wash in 1X PBS, floating sections were mounted with DAPI Fluoromount-G (Electron Microscopy Sciences Cat. #17984–24) or with VectaShield Plus Antifade Mounting Medium with DAPI (Vector Laboratories LOT ZL0420) and sealed with Sally Hansen “Dries Instantly” Topcoat. **Primary antibodies**; Goat anti-ChAT (1:200, AB114P, Millipore), Mouse anti-GAD67 (1:200, MAB5406, Millipore), and Rat anti-cFOS (1:500, 226308, Synaptic Systems). **Secondary antibodies**: Alexa Fluor 488 donkey anti-mouse (1:500, A21202), Alexa Fluor 555 donkey anti-goat (1:500, A21432), and Alexa Fluor 647 donkey anti-rat (1:500, A48272).

### 2.6 Confocal imaging

Mounted sections were imaged using a Leica TCS-SP8 laser scanning confocal microscope with Leica’s LASX acquisition software. Imaging parameters were established using control sections and maintained as Leica sequence files, which were kept constant between conditions to allow for direct quantitative comparison. Z-stacks were acquired for each medial septum (10x magnification, 10 µm thickness, 2 µm steps) by establishing the upper bounds at the most dorsal ChAT+ septal neuron and using the Islands of Calleja (ISC) to differentiate the lower bounds of the MS from the vertical diagonal band of Broca (VDB). LASX was used to generate maximum projections for each MS before the images were loaded into QuPath for analysis.

### 2.7 Cell counting and analysis

QuPath is an open-source software program for image analysis, typically for pathological/histological applications (Bankhead et al. 2017). A QuPath project file was made for each analysis, and the relevant Max-Z images were added before adjusting the brightness and contrast to improve clarity (the quantification is agnostic to these changes). Each medial septum was annotated manually with closed polygons using the aforementioned (**2.6**) anatomical boundaries to account for individual variation. Cells were either detected using the DAPI nuclear counterstain or directly from the respective channel using the “Cell Detection/Positive Cell Detection” function. Cell boundaries were defined with a consistent “expansion” value of 3μm relative to the nucleus, thereby excluding anything outside the soma for quantification. Composite classifiers consisting of a single measurement classifier for each marker were used to confirm the initial findings and for subsequent supplementary data. All thresholding and detection parameters were kept constant within each analysis, and cell counts were standardized to the annotation areas. Raw data from QuPath was copied into Microsoft Excel and organized before being exported into GraphPad Prism 10 software for statistical analysis and figure creation.

## 3 Results

To study the effects of chronic stress, we used the unpredictable chronic mild stress paradigm (uCMS), in which animals are exposed to random environmental and psychological stressors daily to mimic human life stressors (Nollet, 2021). After two weeks of uCMS, we tested animals in the A-B Discrimination Test (Fig. 1) to evaluate the ability of mice to distinguish between two similar contexts, which specifically relies on medial septum and hippocampal circuit function. Discrimination scores, calculated as (freezing time in Context A - Context B) / (freezing time in Context A + B), were used as a measure of context discrimination, with higher scores indicating better neural circuit function.

**Fig. 2:**
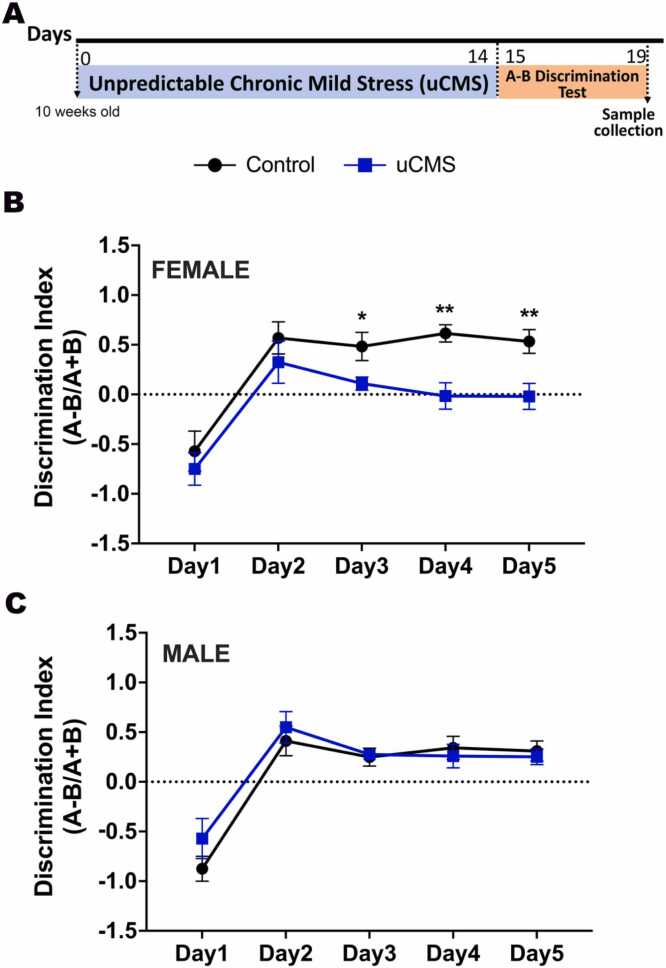
Behavioral effects of uCMS in the A-B Discrimination Test is sex-dependent. A. Experimental timeline. **B.** Female uCMS mice exhibited deficits in discriminating between the shock and no-shock contexts, as reflected by a reduced discrimination index (N = Control: 7, uCMS: 9). **C.** Male mice showed no significant differences in discrimination performance between experimental groups (N = Control: 8, uCMS: 7). Data are presented as mean ± s.e.m. and analyzed using two-way ANOVA. *p < 0.05, **p < 0.01.

**Fig. 3:**
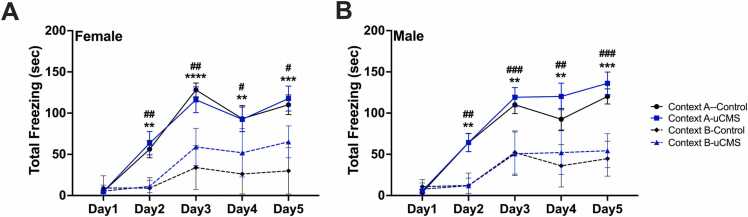
Total freezing scores of control and uCMS females and males in shock-paired Context A and neutral Context B. A. In females, both control and uCMS groups exhibited higher freezing in shocked-paired Context A; however, uCMS females also showed significantly elevated freezing in neutral Context B compared to controls. **B.** In males, both control and uCMS groups displayed increased freezing in Context A and reduced freezing in Context B. Data are presented as mean ± s.e.m. and analyzed using two-way ANOVA. Control Context A vs Context B: **p < 0.01, ***p < 0.001, ****p < 0.0001. uCMS Context A vs Context B: ^#^p < 0.05, ^##^p < 0.01, ^###^p < 0.001.

We tested male and female adult mice in two experimental groups: uCMS and control (not disturbed) (Fig. 2A). Female uCMS mice showed deficits in differentiating the two different contexts compared to female control mice, shown by a lower discrimination index (repeated-measures two-way ANOVA: uCMS: F(1,14)= 11.22, p = 0.005; Days: F(2.735, 35.29)= 20.92, p < 0.0001) (Fig. 2B). Interestingly, uCMS had no significant effect in males; both control and uCMS-exposed groups showed progressive context discrimination over time (repeated-measures two-way ANOVA: Days: F(4, 52)= 33.66, p < 0.0001) (Fig. 2C). Next, we analyze the total freezing time in both contexts separately. In females, control animals displayed significantly higher freezing time in the shock-paired context (Context A) than in the neutral context (Context B) (repeated-measures two-way ANOVA: Context: F(1,12)= 41.59, p < 0.0001; Days: F(2.367, 28.40)= 26.90, p < 0.0001, Days x Context: F(4, 48)= 10.95, p < 0.0001) (Fig. 3A). Female uCMS animals also showed a significant higher freezing time in the shock-paired context (Context A) than in the neutral context (Context B)(repeated-measures two-way ANOVA: Context: F(1,16)= 20.14, p = 0.0005; Days: F(2.609, 41.74)= 30.16, p < 0.0001, Days x Context: F(4, 64)= 3.126, p = 0.0217). Although the total freezing time was not significantly different between control and uCMS females in shock-paired Context A, females in the uCMS group exhibited significantly elevated freezing in the neutral Context B relative to controls (repeated-measures two-way ANOVA: uCMS: F(1,14)= 5.587, p = 0.0343; Days: F(2.107, 29.50)= 19.95, p < 0.0001, Days x uCMS: F(4, 56)= 3.576, p = 0.0119) (Fig. 3A). This elevated freezing in Context B, despite no difference in freezing levels in Context A between groups, suggests that uCMS induces generalized fear in females. In males, both the control (repeated-measures two-way ANOVA: Context: F(1,14)= 37.42, p < 0.0001; Days: F(2.595, 36.32)= 35.59, p < 0.0001, Days x Context: F(4, 56)= 8.659, p < 0.0001) and uCMS (repeated-measures two-way ANOVA: Context: F(1,12)= 23.35, p = 0.0004; Days: F(2.977, 35.72)= 63.14, p < 0.0001, Days x Context: F(4, 48)= 11.70, p < 0.0001) animals exhibited significantly increased freezing time in shock-paired context (Context A) compared to the neutral context (Context B) (Fig. 3B). Freezing time of control and uCMS males were not different from each other neither in Context A nor in Context B (Fig. 3B).

**Fig. 4:**
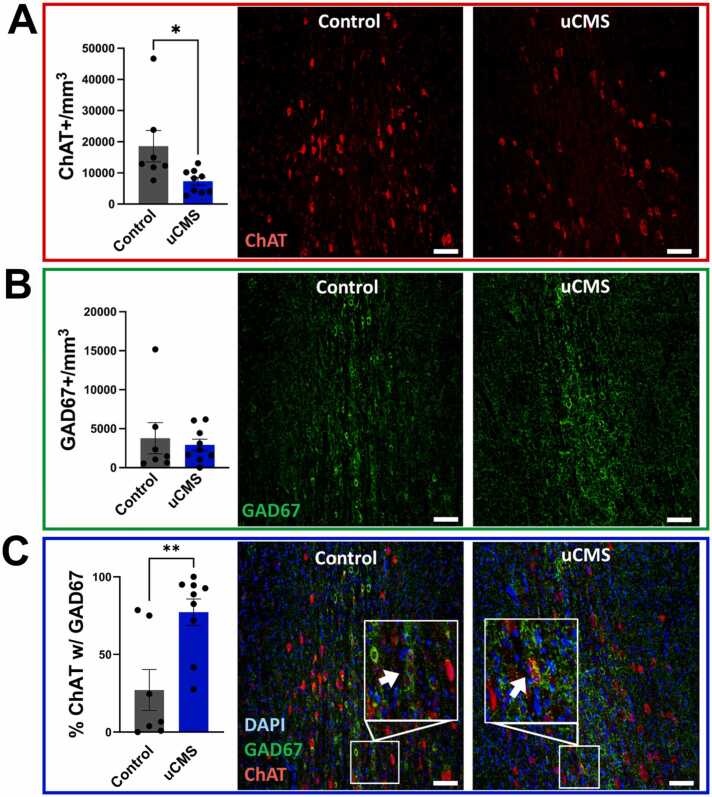
ACh and GABA co-expression in the medial septum of female mice following uCMS. A. uCMS significantly reduced the number of ChAT positive (ChAT+) cholinergic neurons in the medial septum. **B.** The number of GAD67 positive (GAD67 +) GABAergic neurons remained unchanged after uCMS. **C.** uCMS significantly increased the percentage of ChAT+ neurons that also co-expressed GAD67. Scale bar = 50um. Data are presented as mean ± s.e.m. and analyzed using Student’s *t*-test. *p < 0.05, **p < 0.01.

To determine whether uCMS changed acetylcholine (ACh) and GABA expression, we quantified the number of neurons expressing synthetic enzymes for ACh (choline acetyltransferase, ChAT) and GABA (glutamic acid decarboxylase 67, GAD67) in the medial septum. In female mice, two weeks of uCMS significantly reduced ChAT+ neuron numbers (Student’s *t*-test: t(14) = 2.438, p < 0.05) without affecting GAD67 + neurons (Fig. 4A-B), indicating selective vulnerability of ACh neurons to uCMS. Despite this, the percentage of ChAT+ neurons that also expressed GAD67 + increased (Student’s *t*-test: t(14) = 3.312, p < 0.001) (Fig. 4C), with no evidence of apoptosis (Supplemental Figure 1 A). These results suggest that ChAT neurons co-expressing GAD67 may be more resilient to uCMS-induced alterations. Alternatively, some ChAT+ neurons may downregulate cholinergic markers or undergo transmitter switching to express other phenotypes (e.g., VGLUT2), which were not quantified here.

**Fig. 5:**
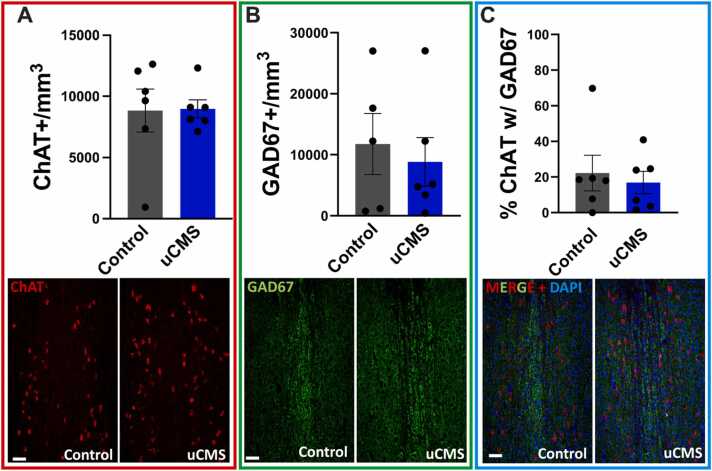
ACh and GABA expression in the medial septum of male mice following uCMS is unchanged. A. uCMS did not alter the number of ChAT positive (ChAT+) cholinergic neurons in the medial septum. **B.** The number of GAD67 positive (GAD67 +) GABAergic neurons remained unchanged after uCMS. **C.** The percentage of ChAT+ neurons expressing GAD67 also showed no significant changes. Data are presented as mean ± s.e.m. and analyzed using Student’s *t*-test.

In contrast, uCMS had no significant effect on the number of ChAT+ or GAD67 + neurons in male mice (Fig. 5A-B), nor did it alter the percentage of ChAT+ neurons expressing GAD67 + (Fig. 5C). These results indicate a sex-specific effect of chronic stress on medial septum neurotransmitter expression, with female mice showing selective reductions in ChAT+ neurons and altered ACh-GABA co-expression, whereas males showed no changes in these measures consistent with their behavioral performance.

**Fig. 6:**
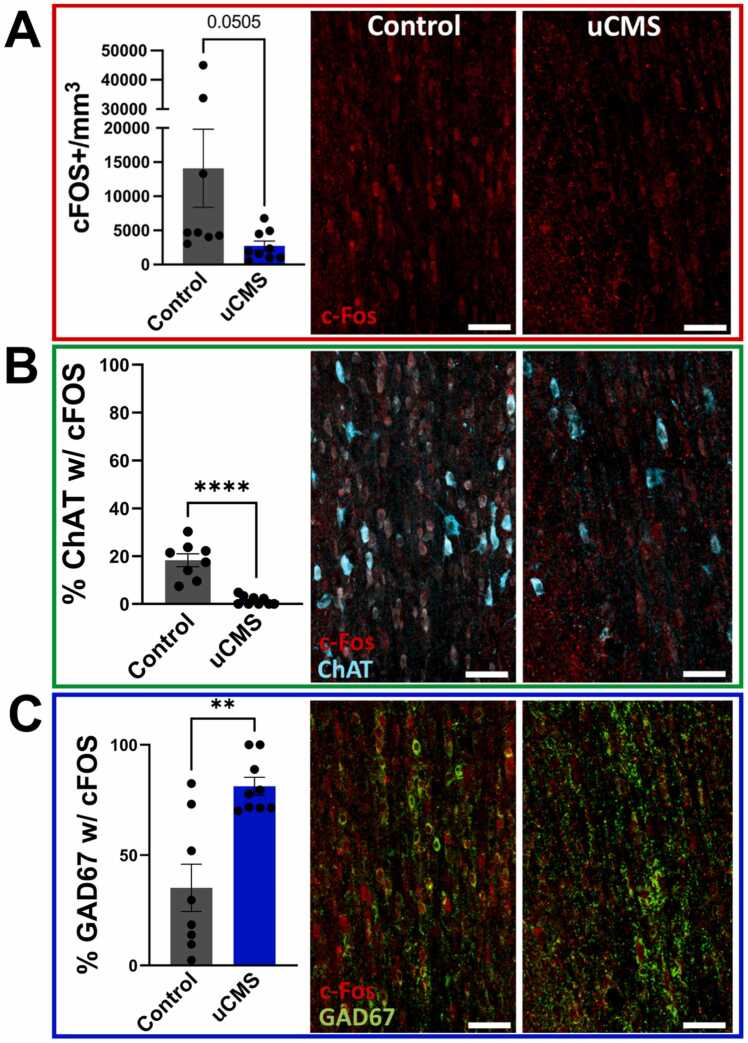
Effects of uCMS on neuronal activity (cFOS expression) in the medial septum of female mice. A. uCMS reduced (p = 0.0505) the total number of cFOS positive (cFOS+) neurons in the medial septum, indicating decreased neuronal activity. **B.** The percentage of ChAT positive (ChAT+) cholinergic neurons that expressed cFOS significantly decreased following uCMS exposure. **C.** The percent of GAD67 positive (GAD67 +) GABAergic neurons that expressed cFOS significantly increased following uCMS. Data are presented as mean ± s.e.m. and analyzed using Student’s *t*-test. **p < 0.01; ****p < 0.0001.

**Fig. 7:**
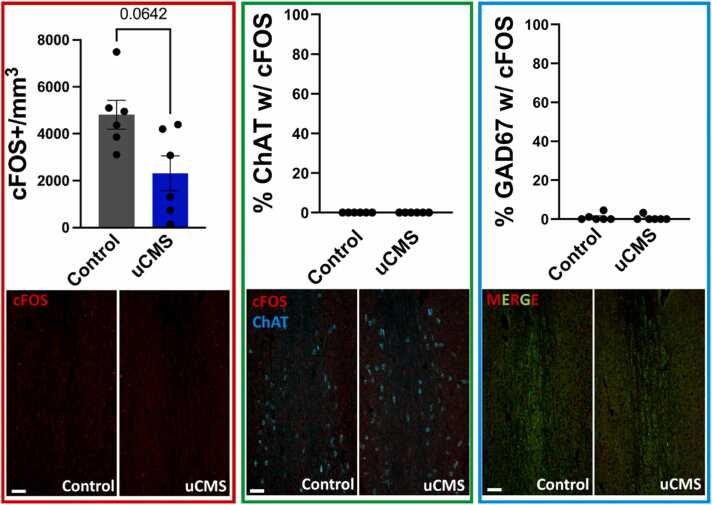
Effects of uCMS on neuronal activity (cFOS expression) in the medial septum of male mice. A. uCMS did not alter the total number of cFOS positive (cFOS+) neurons in the medial septum, indicating no changes in neuronal activity. **B.** cFOS expression in ChAT positive (ChAT+) cholinergic neurons and **C.** in (GAD67 +) GABAergic neurons remained unchanged following uCMS exposure. Data are presented as mean ± s.e.m. and analyzed using Student’s *t*-test.

Neurotransmitter phenotype plasticity is typically regulated by sustained neuronal activity, driven either experimentally or through natural sensory inputs (Agid et al., 2000; Dannenberg et al. 2015; Kiraly et al., 2023; Mamad et al. 2015; Müller and Remy, 2018; Teles-Grilo Ruivo and Mellor, 2013; Wander et al. 2023; Paul et al. 2015; Kniffin et al. 2024). To investigate changes in neuronal activity in different medial septum neuronal populations, we stained brain sections with immediate early gene marker cFOS, a proxy marker for active neurons. In female mice, uCMS led to a modest reduction in overall neuronal activity within the medial septum, as indicated by relatively decreased numbers of cFOS+ neurons (Student’s *t*-test: t(15) = 2.09, p = 0.0505) (Fig. 6A). This reduction was accompanied by significantly diminished activity of ChAT+ neurons, reflected in lower cFOS co-localization with ChAT+ neurons (Student’s *t*-test: t(15) = 6.404, p < 0.0001) (Fig. 6B). Notably, despite the near-global reduction in activity and suppressed ChAT+ neuronal activity, uCMS selectively increased the activity of GAD67 + neurons, as evidenced by a higher percent of cFOS+ , GABAergic neurons (Student’s *t*-test: t(15) = 4.196, p < 0.001) (Fig. 6C). By contrast, male mice showed no significant changes in cFOS+ , ChAT+ & cFOS+ , or GAD67 + & cFOS+ neuron counts following uCMS (Fig. 7), indicating resilience to stress-induced alterations in medial septum neuronal activity. Compared to females, male mice exhibited decreased overall cFOS expression in the medial septum (Fig. 6A & 7). This difference is largely driven by a small number of high-cFOS female mice, as the majority of the samples (males and females) had overall cFOS cell counts between 3000 and 5000/mm^3^ in the medial septum. The difference is far more pronounced when looking specifically at both ChAT+ and GAD67 + neurons, which exhibited little-to-no cFOS expression for either condition in males (Fig. 7). These findings highlight a sex-specific response, with uCMS shifting the balance of medial septum activity in females, suppressing cholinergic (ChAT+) activity while enhancing GABAergic (GAD67 +) activity, and potentially contributing to disruptions in ACh-GABA co- expression.

## 4 Discussion

Our study establishes stress-induced alterations in neurotransmitter co-expression as a potential mechanism through which chronic stress disrupts medial septum function and hippocampal-dependent behavior. Using the unpredictable chronic mild stress (uCMS) paradigm, we demonstrate that stress drives a maladaptive shift in ACh-GABA co-expression in medial septum neurons, leading to cognitive deficits in a sex-specific manner; we observed these changes exclusively in female mice, underscoring the central roles of both sex and co-expression changes in stress-induced dysfunction.

The key finding is that uCMS reduces the number of cholinergic (ChAT+) neurons while increasing the proportion of cholinergic neurons that co-express GABA (GAD67 +) in the female mouse medial septum. Because the overall number of GAD67 + neurons remained unchanged, this increase in co-expression likely reflects either a selective resilience of ChAT neurons that also express GAD67 or a downregulation of ChAT expression in a subset of cholinergic neurons. Another possibility is that some ChAT+ neurons may undergo neurotransmitter switching to express alternative markers such as VGLUT2, which were not quantified here. Regardless of the mechanism, these changes are likely maladaptive, as they coincide with impairments to context discrimination, potentially due to disruptions in the excitatory-inhibitory balance within hippocampal circuits. The reduction in septal ChAT+ neurons likely decreases cholinergic input to the hippocampus, and the relative overrepresentation of GABAergic signaling withing this diminished cholinergic system could perturb circuit function and contribute to the observed deficits in hippocampal-dependent behavior. This finding aligns with prior work showing that perturbations in septal cholinergic tone disrupt hippocampal oscillations and impair spatial and episodic memory (Kniffin et al. 2024). Our data extend these findings by revealing that not just the presence but the neurotransmitter identity of MS neurons, specifically their co-expression profile, may play a critical role in cognitive outcomes under chronic stress.

Accompanying the changes in co-expression, uCMS moderately suppresses MS neuronal activity in females as measured by cFOS expression, primarily through reduced activity of cholinergic ChAT+ neurons (*highly* significant). In contrast, female GAD67 + GABAergic neurons exhibit increased activity, further tilting the balance toward GABA-driven inhibition. Male mice showed no significant differences in neuronal activity, cFOS expression, between conditions but *did* exhibit markedly lower cFOS expression in both ChAT+ and GAD67 + neurons than females. Differences in overall cFOS expression between sexes were driven largely by high female variability and may not necessarily indicate baseline differences in medial septum neuron activity. The stark sex differences in cFOS expression within cholinergic and GABAergic neurons raise the possibility that males recruit alternative neuronal populations to regulate medial septum function under stress. Indeed, glutamatergic neurons constitute a fraction of the medial septum and have been shown to be engaged in the development of PTSD-like symptoms (Wu et al., 2025). Lower cFOS in ChAT+ and GAD67 + neurons in males may therefore be accompanied by preferential engagement of glutamatergic medial septum neurons. Another possibility is the recruitment of other brain regions that may compensate for weaker medial septum cholinergic-GABAergic engagement in males. Such compensatory or alternative circuit recruitment may underlie sex-specific strategies for stress adaptation, where females rely more on medial septum cholinergic-GABAergic signaling and males recruit parallel or extra-septal circuits. We will address these possibilities in our future studies.

It is known that neuronal activity mediates neurotransmitter identity changes and is necessary to maintain the newly acquired transmitter phenotype (Spitzer, 2015, Spitzer, 2017). This process suggests that stress-induced hyperactivity of GAD67 + neurons may promote a lasting shift in neurotransmitter co-expression. This shift could reflect a broader failure of homeostatic plasticity in stressed animals, where compensatory mechanisms such as increased inhibitory output exacerbate network instability. Notably, medial septum GABAergic projections are essential for maintaining theta rhythm synchrony (Hangya et al. 2009), and excessive inhibition can disrupt hippocampal theta oscillations critical for memory encoding and retrieval (Kota et al. 2020). Over time, this maladaptive shift could diminish the flexibility of medial septum output, leading to persistent deficits in hippocampal theta rhythm generation and downstream disruptions in neurogenesis and synaptic plasticity. In this context, the observed cognitive impairments may arise from circuit-level rigidity, a failure to dynamically adjust neuromodulatory tone in response to environmental demands. While we did not directly investigate the long-term stability of these transmitter phenotype changes or their broader effects on hippocampal circuitry, these mechanisms warrant further study to elucidate their contribution to cognitive deficits and circuit dysfunction in stress-related neuropsychiatric disorders.

Importantly, these findings highlight the sex-specific nature of co-expression dysregulation. While females exhibit clear vulnerability to uCMS, male mice remain resilient, showing no significant changes in ACh-GABA co-expression, MS neuronal activity, or hippocampal-dependent behavior. Sex differences in estrogen signaling likely contribute to this disparity. Estrogen is a key modulator of both cholinergic and GABAergic systems. It supports cholinergic neuron function by increasing neuronal activity (Szegő et al. 2006), enhancing expression of the acetylcholine-synthesizing enzyme ChAT (Pongrac et al. 2004), and improving cognitive performance (Newhouse & Dumas, 2015), further supporting the hypothesis that the cholinergic system is a major site of estrogen action in the brain. In parallel, estrogens regulate GABAergic transmission by modulating gene expression (McCarthy et al., 1995, Herbison, 1997), GABA receptor subunit composition (Herbison & Fenelon, 1995), and GABAergic neurotransmission (Mukherjee et al. 2017); thereby contributing to excitatory-inhibitory balance in stress-sensitive circuits. These effects of estrogen signaling on both cholinergic and GABAergic systems highlight its potential role in regulating ACh-GABA co-transmission. Moreover, cognitive-enhancing effects of estrogen have been demonstrated in both preclinical and clinical studies (Russell et al., 2019), where it facilitates learning, memory consolidation, and hippocampal synaptic plasticity. Additionally, the role of estrogen in mood regulation is supported by evidence that estrogen treatment can alleviate depressive symptoms and enhance antidepressant efficacy (Sun et al. 2024). Given these cognitive- and mood-enhancing effects and regulatory role of estrogen on cholinergic and GABAergic systems, the influence of estrogen on neurotransmitter co-expression may underlie individual differences in stress susceptibility and resilience. Understanding this interaction may yield mechanistic insight into the neural basis of stress-related disorders and inform sex-specific therapeutic strategies.

Further, females have been shown to exhibit enhanced basal levels of neuroinflammation (Osborne et al. 2018) and differential microglial activation (Bollinger et al. 2016), reflecting a fundamentally different neuroimmune landscape compared to males. Stress further amplifies these sex-specific responses by increasing microglial reactivity (Schramm & Waisman, 2022) and elevating pro-inflammatory cytokine production (Alotiby, 2024), which in turn can alter synaptic remodeling, neurotransmitter dynamics, and circuit excitability. Notably, the cholinergic system plays a pivotal role in regulating neuroimmune function through the “cholinergic anti-inflammatory pathway,” in which acetylcholine suppresses inflammatory signaling via α7 nicotinic receptors on immune cells (Gallowitsch-Puerta and Pavlov, 2007). Microglia themselves express cholinergic receptors and respond to local cholinergic tone, creating a direct interface between neural activity and immune state (Nazmi et al. 2021). When this regulatory axis is disrupted under chronic stress, impaired cholinergic control of microglia may lead to persistent inflammation, perturbations in ACh-GABA co-expression, and destabilization of stress-sensitive circuits. These maladaptive changes are likely to be more pronounced in females due to their enhanced neuroimmune sensitivity, helping to explain their increased susceptibility to mood and stress-related disorders.

In conclusion, our study identifies stress-induced alterations in ACh-GABA co-expression as a key mechanism linking medial septum dysfunction to hippocampal-dependent cognitive deficits, specifically in females. These findings establish that co-expression is not merely a static feature of cholinergic neurons, but a dynamic and stress-sensitive process that can shift toward maladaptive states under chronic challenge. The observed imbalance between excitatory cholinergic and inhibitory GABAergic signaling provides a mechanistic framework for understanding how stress undermines medial septum output and disrupts hippocampal function. Notably, the female-specific nature of these changes suggests that sex hormones or heightened neuroinflammatory responses may influence transmitter identity and plasticity in stress-sensitive circuits, raising important questions about the hormonal and molecular drivers of co-expression reprogramming. This work advances our understanding of neurotransmitter phenotype plasticity as a modifiable contributor to stress vulnerability and suggests that restoring transmitter balance in medial septum neurons may represent a novel therapeutic strategy for improving cognitive outcomes in stress-related neuropsychiatric disorders.

## CRediT authorship contribution statement

**Cristina Coffman:** Writing – review & editing, Validation, Investigation. **Anthony Tomaziefski:** Writing – review & editing, Validation, Resources, Methodology, Investigation. **Elif Tunc-Ozcan:** Writing – review & editing, Writing – original draft, Visualization, Validation, Supervision, Software, Resources, Project administration, Methodology, Investigation, Funding acquisition, Formal analysis, Data curation, Conceptualization. **Renae Simonson:** Writing – review & editing, Validation, Investigation. **Christopher Mazon:** Writing – review & editing, Writing – original draft, Resources, Project administration, Methodology, Investigation, Formal analysis, Conceptualization. **Gabriella Salmeron-Ceballos:** Writing – review & editing, Validation, Resources, Methodology, Investigation. **Betters Ryan Kenly:** Writing – review & editing, Writing – original draft, Visualization, Validation, Supervision, Investigation, Formal analysis, Data curation.

## Compliance with ethical standards

All procedures were performed in compliance with the Institutional Animal Care and Use Committee (IACUC) under protocol #23–201412-HSC, USDA # 85-R-0014, and Animal Welfare Assurance # D16–00228 (A3350–01); amended 12/05/24.

## Funding

This work was supported by the National Institutes of Health: National Institute for Mental Health [NIMH R00-MH1250155] and 10.13039/100000057National Institute of General Medical Sciences [NIGMS 2P20GM109089].

## Declaration of Competing Interest

None.

## Footnotes

Appendix A Supplementary data associated with this article can be found in the online version at doi:10.1016/j.ibneur.2026.01.003.
