# Supplementary material for: Chronic stress alters neurotransmitter co-expression and disrupts context discrimination in a sex-dependent manner
Source: IBRO Neurosci Rep. 2026 Jan 6;20:160–9. doi: 10.1016/j.ibneur.2026.01.003 (PMC12856636; doi:10.1016/j.ibneur.2026.01.003)
Supplement: Supplementary file 1 — Supplementary material [file mmc1.docx]

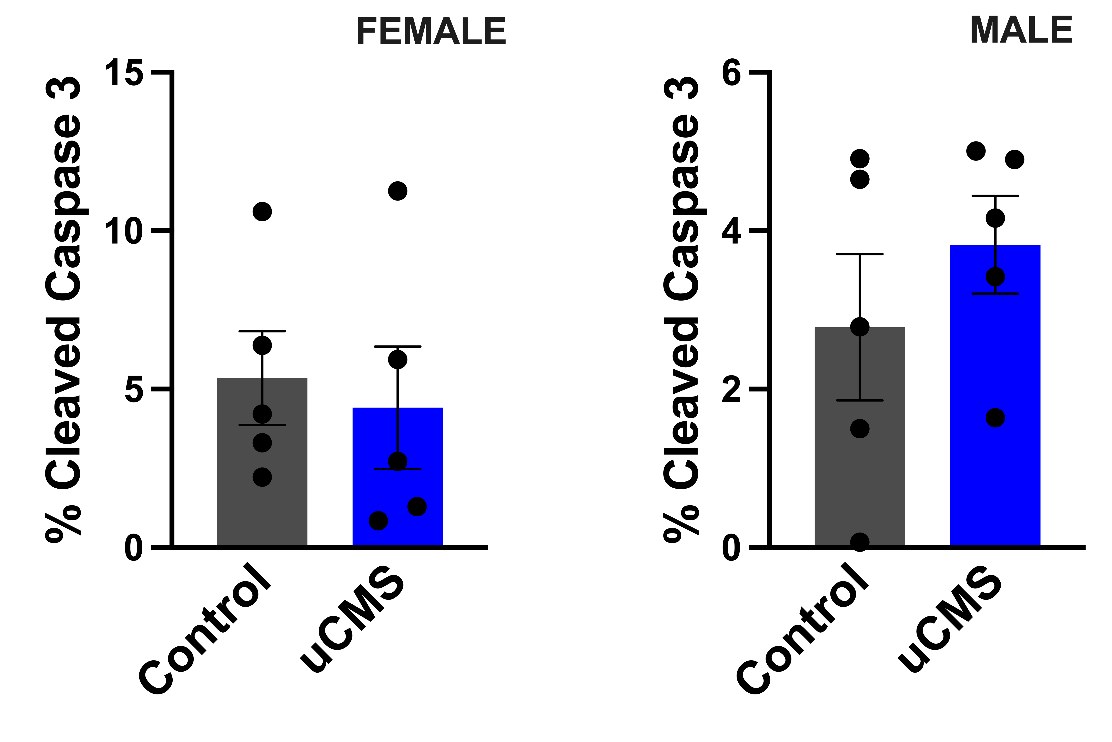


**Supplemental Figure 1.** Apoptotic activity, as shown by cleaved caspase 3 immunoreactivity, in the medial septum does not differ significantly between control and uCMS mice for neither males nor females.
